# Hierarchical Cache-Aided Networks for Linear Function Retrieval

**DOI:** 10.3390/e26030195

**Published:** 2024-02-25

**Authors:** Lingyu Zhang, Yun Kong, Youlong Wu, Minquan Cheng

**Affiliations:** 1 Guangxi Key Laboratory of Multi-Source Information Mining & Security, Guangxi Normal University, Guilin 541004, China; zhangmm@stu.gxnu.edu.cn; 2 The Department of Electrical Engineering, University of North Texas, Denton, TX 76207, USA; yunkong@my.unt.edu; 3 The School of Information Science and Technology, ShanghaiTech University, Shanghai 201210, China

**Keywords:** linear function retrieval, hierarchical coded caching scheme, transmission load

## Abstract

In a hierarchical caching system, a server is connected to multiple mirrors, each of which is connected to a different set of users, and both the mirrors and the users are equipped with caching memories. All the existing schemes focus on single file retrieval, i.e., each user requests one file. In this paper, we consider the linear function retrieval problem, i.e., each user requests a linear combination of files, which includes single file retrieval as a special case. We propose a new scheme that reduces the transmission load of the first hop by jointly utilizing the two layers’ cache memories, and we show that our scheme achieves the optimal load for the second hop in some cases.

## 1. Introduction

In order to reduce the transmission pressure of wireless networks during peak traffic times, Maddah-Ali and Niesen in [1] provided a (K, M, N) coded caching scheme (MN Scheme) where a single server has *N* files and connects *K* cache-aided users with the cache memories of *M* files through an error-free shared link. A coded caching scheme consists of two phases: (1) the placement phase, where the server is equipped with the data and each user’s cache is also equipped with the size of at most *M* files without knowledge of the users’ future demands; (2) the delivery phase, where each user randomly requests one file and then the server sends the coded signal to the users such that each user can decode its requested file with the help of its cached packets. It is shown that the MN Scheme is generally order-optimal within a factor of 2 [2] and optimal under the uncoded data placement when K≤N [3]. The MN Scheme is also widely used in different networks, such as combination networks [4,5], device-to-device networks [6], etc.

**Figure 1:**
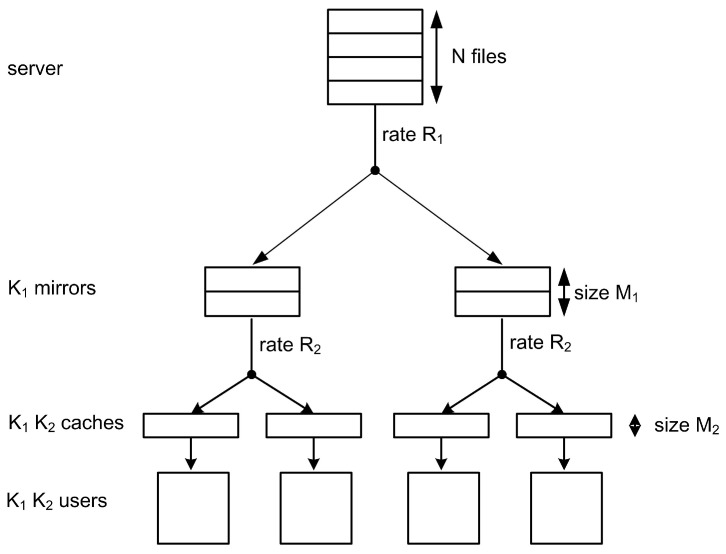
The $\left(K_{1},\;K_{2};\;M_{1},\;M_{2};\;N\right)$ hierarchical caching system with N=4, $K_{1}\;=\;K_{2}\;=\;2$, $M_{1}\;=\;2$, and $M_{2}\;=\;1$.

In practical scenarios, caching systems are transformed into multiple layers in order to make transmission more efficient, such as the hierarchical edge caching architecture for Internet of Vehicles [7], the three-tier mobile cloud-edge computing structure [8], and so on. In this paper, we particularly study the hierarchical caching system [9], a two-layer network as illustrated in Figure 1. A $(K_{1}$, $K_{2}$; $M_{1}$, $M_{2}$; N) hierarchical caching system consists of a single server with a library of *N* files, $K_{1}$ cache-aided mirror sites, and $K_{1}K_{2}$ cache-aided users. For the first layer, the $K_{1}$ mirror sites are connected to the server through an error-free shared link, and for the second layer, each user connects to only one mirror. Our goal is to design a scheme to decrease the first load $R_{1}$ in the first hop (i.e., from the server to all the mirror sites) and the second load $R_{2}$ in the second hop (i.e., from each mirror site to its connected users).

The authors in [9] proposed the first hierarchical coded caching scheme (KNMD Scheme). The MN Scheme is applied two times in two layers consecutively. Although the KNMD Scheme achieves the optimal transmission load for the second hop, it involves a significant increase in $R_{1}$ since it ignores the users’ cache memory when designing the multicast message sent from the server. To improve the first load $R_{1}$, the authors in [10,11] proposed new schemes that jointly use the two types of the MN Scheme together for the mirror sites and users, respectively.

It is worth noting that all the schemes consider the single file retrieval case, i.e., each user requests one file. The authors in [12] first considered the linear function retrieval scheme (WSJT Scheme), i.e., a linear combination of files is requested from each user through the shared link broadcast network. Clearly, linear function retrieval includes the single file retrieval case. In this paper, we study the linear function retrieval scheme for hierarchical networks and obtain the following results.

- We first propose a baseline scheme via the WSJT Scheme and KNMD Scheme where the second-layer load achieves the optimal transmission load. However, we achieve this by sacrificing the first-layer load.
- Then, in order to reduce the first-layer load, we propose another scheme whose second load also achieves optimality at the expense of increased subpacketization. Our scheme also aids in reducing the redundancy for some special demand distributions.

The rest of this paper is organized as follows. Section 2 formally introduces the system model and some existing schemes. Section 3 presents the main results. Section 4 gives an example and the general description of our scheme, i.e., the scheme for Theorem 2. The conclusion of this paper is given in Section 5.

Notations: For any positive integers *a* and *b* with a<b, let [a:b]≜{a,…,b} and [a]≜[1:a]. Let [b]t≜{V|V⊆[b],|V|=t}, for any positive integer t≤b. For a positive integer *n*, the n-dimensional vector space over the field $F_{q}$ is denoted by $F_{q}^{n}$. For a given matrix P with row size $X_{1}$, we divide it into $X_{1}$ parts by row, which is represented by $P\;=\;\{P^{\left(x_{1}\right)}|\;x_{1}\;\in \;\left[X_{1}\right]\}$. For any integer set T, define $P_{T}$ as the sub-matrix of P by selecting some rows from P, where the rows have indices in T. The rank of matrix P is denoted as rank(P). The transpose of P is represented by $P^{⊤}$.

## 2. Preliminary

In this section, we give a formal description of the hierarchical caching system and review some existing related schemes for the hierarchical caching problem.

### 2.1. System Model

Consider a hierarchical network as shown in Figure 1. It consists of a single server with a library of *N* files, $K_{1}$ cache-aided mirror sites, and $K_{1}K_{2}$ cache-aided users. For the first layer, the $K_{1}$ mirror sites are connected to the server through an error-free shared link, and for the second layer, each user connects to only one mirror. M_*k*1_ represents the $k_{1}$-th mirror and the $k_{2}$-th user attached to $M_{k_{1}}$ as $U_{k_{1},k_{2}}$, $k_{1}\;\in \;\left[K_{1}\right],\;k_{2}\;\in \;\left[K_{2}\right]$, and the set of users attached to $M_{k_{1}}$ as $U_{k_{1}}$. The server contains a collection of *N* files, denoted by $W\;=\;\{W^{\left(1\right)},W^{\left(2\right)},\ldots ,W^{\left(N\right)}\}$, each of which is uniformly distributed over $F_{2}^{B}$, where $B\;\in \;N^{+}$. Each mirror and user is equipped with $M_{1}$ and $M_{2}$ files, respectively, where $M_{1}$, $M_{2}\geq 0$. A $\left(K_{1},\;K_{2};\;M_{1},\;M_{2};\;N\right)$ hierarchical caching system contains two phases.

- **Placement phase:** The mirror site M_*k*1_ caches some parts of the files by using a cache function $\varphi_{k_{1}}\;:\;F_{2}^{NB}\;\;\to \;\;F_{2}^{M_{1}B}$, where $\frac{M_{1}}{N}$ is the memory ratio of the mirror in the first layer, $M_{1}\;\in \;[0:N]$. The cache contents of mirror $M_{k_{1}}$ are
  $$Z_{k_{1}}\;=\;\varphi_{k_{1}}\left(W\right),\;k_{1}\;\in \;\left[K_{1}\right].$$
  The user $U_{k_{1},k_{2}}$ caches some parts of the files by using a cache function $\phi_{k_{1},k_{2}}\;:\;F_{2}^{NB}\;\;\to \;\;F_{2}^{M_{2}B}$, where $\frac{M_{2}}{N}$ is the memory ratio of users in the second layer, $M_{2}\;\in \;[0:N]$. Then, the cache contents of $U_{k_{1},k_{2}}$ are
  $${\tilde{Z}}_{k_{1},k_{2}}\;=\;\phi_{k_{1},k_{2}}\left(W\right),k_{1}\;\in \;\left[K_{1}\right],\;k_{2}\;\in \;\left[K_{2}\right].$$
- **Delivery phase:** Each user $U_{k_{1},k_{2}}$ randomly requests a linear combination of the files
  $$L_{k_{1},k_{2}}\;=\;d_{k_{1},k_{2}}^{\left(1\right)}W^{\left(1\right)}+d_{k_{1},k_{2}}^{\left(2\right)}W^{\left(2\right)}+\ldots +d_{k_{1},k_{2}}^{\left(N\right)}W^{\left(N\right)}.$$
  for any $k_{1}\;\in \;\left[K_{1}\right],\;k_{2}\;\in \;\left[K_{2}\right]$, where $d_{k_{1},k_{2}}\;=\;(d_{k_{1},k_{2}}^{\left(1\right)},\ldots$, $d_{k_{1},k_{2}}^{\left(N\right)})\;\in \;F_{2}^{N}$ denotes the demand vector of user $U_{k_{1},k_{2}}$. When each user requests a single file, the demand vector $d_{k_{1},k_{2}}$ is a *N*-length unit vector. For example, if user $U_{k_{1},k_{2}}$ only requests the 1-st file, then the demand vector is set as $d_{k_{1},k_{2}}\;=\;\left(1,\ldots ,0\right)\;\in \;F_{2}^{N},k_{1}\;\in \;\left[K_{1}\right],k_{2}\;\in \;\left[K_{2}\right]$, which is a special case of our proposed scheme. We can obtain the demand matrices of all users as follows:
  (1) $$D^{\left(k_{1}\right)}\;=\;\left(\begin{array}{c} d_{k_{1},1}\\ \vdots \\ d_{k_{1},K_{2}} \end{array}\right),\;\;\;\;\;D\;=\;\left(\begin{array}{c} D^{\left(1\right)}\\ \vdots \\ D^{\left(K_{1}\right)} \end{array}\right).$$
  where $D^{\left(k_{1}\right)}$ represents the demand vectors of $\;U_{k_{1}}$. Given the demand matrix D, we should consider the following two types of messages.
  – **The messages sent by the server:** The server generates signal $X^{\mathrm{server}}$ by using an encoding function $\chi :F_{2}^{K_{1}K_{2}N}\times F_{2}^{NB}\;\;\to \;\;F_{2}^{R_{1}B}$, where
    $$X^{\mathrm{server}}\;=\;\chi \left(D,W\right).$$
    and then the server sends $X^{\mathrm{server}}$ to the mirrors. The normalized number of transmissions $R_{1}$ is called the transmission load for the first layer.
  – **The messages sent by the mirror:** Based on $X^{\mathrm{server}}$, $Z_{k_{1}}$, and D, each mirror $M_{k_{1}}$ generates a signal $X_{k_{1}}^{\mathrm{mirror}}$ by using the encoding function $\kappa :F_{2}^{K_{2}N}\times F_{2}^{M_{1}B}\times X^{\mathrm{server}}\;\;\to \;\;F_{2}^{R_{2}B}$, where
    $$X_{k_{1}}^{\mathrm{mirror}}\;=\;\kappa \left(D^{\left(k_{1}\right)},Z_{k_{1}},X^{\mathrm{server}}\right).$$
    and then mirror $M_{k_{1}}$ sends $X_{k_{1}}^{\mathrm{mirror}}$ to its connected users. The normalized number of transmissions $R_{2}$ is called the transmission load for the second layer.
  For the retrieval process, each user $U_{k_{1},k_{2}}$ can decode its required linear combination of files from $\left(D,{\tilde{Z}}_{k_{1},k_{2}},X_{k_{1}}^{\mathrm{mirror}}\right)$, which means that there exist decoding functions $\xi_{k_{1},k_{2}}:F_{2}^{K_{1}K_{2}N}\times F_{2}^{M_{2}B}\times F_{2}^{R_{2}B}\;\;\to \;\;F_{2}^{B}$, $k_{1}\;\in \;\left[K_{1}\right],k_{2}\;\in \;\left[K_{2}\right]$, such that
  $$\xi_{k_{1},k_{2}}\left(D,{\tilde{Z}}_{k_{1},k_{2}},X_{k_{1}}^{\mathrm{mirror}}\right)\;=\;d_{k_{1},k_{2}}^{\left(1\right)}W^{\left(1\right)}+\ldots +d_{k_{1},k_{2}}^{\left(N\right)}W^{\left(N\right)}.$$

We define the optimal transmission loads for the two layers as $R_{1}^{*}$ and $R_{2}^{*}$ separately.

$$\begin{array}{ccc} R_{1}^{*} & = & \inf_{\chi ,\kappa ,{\left(\xi_{k_{1},k_{2}}\right)}_{k_{1}\in \left[K_{1}\right],k_{2}\in \left[K_{2}\right]}}\left\{R_{1}\right\},\\ R_{2}^{*} & = & \inf_{\chi ,\kappa ,{\left(\xi_{k_{1},k_{2}}\right)}_{k_{1}\in \left[K_{1}\right],k_{2}\in \left[K_{2}\right]}}\left\{R_{2}\right\}. \end{array}$$

Our goal is to design schemes in which the transmission loads $R_{1}$ and $R_{2}$ are as small as possible.

### 2.2. Existing Schemes

In the following, we review the KNMD Scheme for the hierarchical caching problem and the WSJT Scheme over the binary field $F_{2}$, which will be useful for the hierarchical caching system with linear function retrieval. First, let us outline the MN Scheme.

(1) MN Scheme [1]: Set t≜MK/N, when t∈[0:K], N≥K, each file is partitioned into F=Kt packets, i.e., for each n∈[N], $W^{\left(n\right)}\;=\;\left(W_{T}^{\left(n\right)}\right)$, where T∈[K]t. In the placement phase, for each user U_*k*_, k∈[K]. The cache content of user U_*k*_ is Zk={WT(n)|n∈[N],k∈T,T∈[K]t}. In the delivery phase, the file $W_{d_{k}}$ is requested by each user U_*k*_, where $d_{k}\;\in \;\left[N\right]$. Fixing a user k∈S, the user *k* requests the subfiles $W_{d_{k},S\setminus \left\{k\right\}}$ when it is presented in the cache of any user $k^{\prime }\;\in \;S\setminus \left\{k\right\}$. Then, the server transmits the coded signal ${⨁}_{k\in S}W_{d_{k},S\setminus \left\{k\right\}}$, where S⊆[K] of |S|=t+1. The transmission load $R_{\mathrm{MN}}\;=\;\frac{K(1-M/N)}{KM/N+1}$.

(2) KNMD Scheme [9]: This scheme uses the MN Scheme in each layer of the hierarchical network. More specifically, for the first layer between the server and $K_{1}$ mirrors, it uses the $\left(K_{1},M_{1},N\right)$ MN Scheme $K_{2}$ times to recover all $K_{1}K_{2}$ requested files, and then each mirror $M_{k_{1}}$, $k_{1}\in \left[K_{1}\right]$ works as a server whose library contains $K_{2}$ files that are requested by users in $U_{k_{1}}$, and finally it utilizes the $\left(K_{2},M_{2},N\right)$ MN Scheme between $M_{k_{1}}$ and $U_{k_{1}}$. Then, each user can retrieve its requested file with the transmission load as follows.

$$\begin{array}{c} R_{1}=K_{2}\frac{K_{1}-K_{1}M_{1}/N}{K_{1}M_{1}/N+1},\;\;R_{2}=\frac{K_{2}-K_{2}M_{2}/N}{K_{2}M_{2}/N+1}. \end{array}$$

However, the MN Scheme only works for single file retrieval. The authors in [12] proposed a scheme (WSJT Scheme) that is suitable for the linear function retrieval problem.

(3) WSJT Scheme [12]: Using the placement strategy of the MN Scheme, each user U_*k*_ where k∈[K] requests a linear combination of files with demand vector $d_{k}\;\in \;F_{2}^{N}$. After revealing the demand matrix $D\;=\;{\left(d_{1}^{⊤},\ldots ,d_{K}^{⊤}\right)}^{⊤}$ with dimension K×N, the server broadcasts some coded packets by modifying the transmission strategy of the MN Scheme such that each user is able to recover its demanded linear combination of files with the transmission load

RWSJT=Kt+1−K−rank(D)t+1Kt,t∈[0:K].

It is worth noting that when D is row full rank, $R_{\mathrm{WSJT}}$ is optimal under the uncoded placement.

## 3. Main Results

In this section, we first propose a baseline scheme via the WSJT Scheme where $R_{2}$ achieves optimality when the sub-matrix $D^{\left(k_{1}\right)}$, $k_{1}\;\in \;\left[K_{1}\right]$, is full rank. Then, we propose another scheme that improves $R_{1}$ while the $R_{2}$ remains unchanged compared with the Baseline Scheme. Finally, some theoretical and numerical comparisons are provided.

For the sake of convenience in proposing another scheme for some special demand distributions, the following definitions of the leader mirror and user sets are necessary.

**Definition** **1** (Leader mirror set)**.** *For a $K_{1}K_{2}\times N$ demand matrix D in *(1)*, we call a subset of mirrors the leader mirror set, which is represented by $L_{M}\;=\;\left\{l_{1},\ldots ,l_{|L_{M}|}\right\}$, $L_{M}\subseteq \left[K_{1}\right]$, if it satisfies the following condition for $k_{1}\;\in \;\left[K_{1}\right],\;k_{2}\;\in \;\left[K_{2}\right]$*

(2) $$\begin{array}{c} d_{k_{1},k_{2}}\;=\;\alpha_{1}^{\left(k_{1}\right)}d_{l_{1},k_{2}}+\ldots +\alpha_{|L_{M}|}^{\left(k_{1}\right)}d_{l_{|L_{M}|},k_{2}}. \end{array}$$

*and it has the minimum cardinality among all the subsets satisfying *(2)*, where $\left(\alpha_{1}^{\left(k_{1}\right)},\ldots ,\alpha_{|L_{M}|}^{\left(k_{1}\right)}\right)\;\in \;F_{2}^{|L_{M}|}$.*

**Definition** **2** (Leader user set)**.** *For a $K_{2}\times N$ demand matrix $D^{\left(k_{1}\right)}$ in *(1)*, we call a subset of users the leader user set, which is represented by $L_{k_{1}}\;=\;\left\{l_{1}^{\prime },\ldots ,l_{|L_{k_{1}}|}^{\prime }\right\}$, $L_{k_{1}}\subseteq \left[K_{2}\right]$, if, for any $k_{1}\;\in \;\left[K_{1}\right],\;k_{2}\;\in \;\left[K_{2}\right]$, it satisfies the condition *(3)* and it has the minimum cardinality among all the subsets satisfying *(3)*, where $\left(\alpha_{1},\ldots ,\alpha_{|L_{k_{1}}|}\right)\;\in \;F_{2}^{|L_{k_{1}}|}$:*

(3) $$\begin{array}{c} d_{k_{1},k_{2}}\;=\;\alpha_{1}d_{k_{1},1}+\ldots +\alpha_{|L_{k_{1}}|}d_{k_{1},l_{|L_{k_{1}}|}^{\prime }}. \end{array}$$

Now, we introduce the Baseline Scheme, which is generated by using the KNMD Scheme in [9] and the WSJT Scheme in [12]. We utilize the WSJTC Scheme to replace the MN Scheme in the KNMD Scheme, and then we obtain the Baseline Scheme, which is suitable for the linear function retrieval problem in the hierarchical network.

**Theorem** **1** (Baseline Scheme)**.** *For any positive integers $K_{1}$, $K_{2}$, $t_{1}\;\in \;\left[K_{1}\right]$, $t_{2}\;\in \;\left[K_{2}\right]$ and the demand matrix D in *(1)*, there exists a $\left(K_{1},\;K_{2};\;M_{1},\;M_{2};\;N\right)$ hierarchical coded caching scheme for a linear function retrieval problem with memory ratios $\frac{M_{1}}{N}\;=\;\frac{t_{1}}{K_{1}}$, $\frac{M_{2}}{N}\;=\;\frac{t_{2}}{K_{2}}$ and transmission loads*

Rbase1=K2K1t1+1−K1−|LM|t1+1/K1t1.

Rbase2=maxk1∈[K1]K2t2+1−K2−rank(D(k1))t2+1/K2t2.

*where $L_{M}$ is defined in Definition 1.*

In fact, the transmission loads are related to the placement strategy and demand distribution, respectively. The KNMD Scheme considers the first and second layers separately and ignores the users’ and mirrors’ cache memories, which leads to good performance on $R_{2}$ but results in a large transmission load $R_{1}$. For the second layer, it can be regarded as a $\left(K_{2},M_{2},N\right)$ shared link caching problem in which the WSJT Scheme achieves the optimal transmission load under certain circumstances, i.e., when the sub-matrix $D^{\left(k_{1}\right)}$, $k_{1}\;\in \;\left[K_{1}\right]$, is full rank, $R_{\mathrm{base}2}=R_{2}^{*}$. For the purpose of improving $R_{1}$, we propose another scheme, stated below, and the proof is included in Section 4.

**Theorem** **2.** *For any positive integers $K_{1}$, $K_{2}$, $t_{1}\;\in \;\left[K_{1}\right]$, $t_{2}\;\in \;\left[K_{2}\right]$ and the demand matrix D in *(1)*, there exists a $\left(K_{1},\;K_{2};\;M_{1},\;M_{2};\;N\right)$ hierarchical coded caching scheme for a linear function retrieval problem with memory ratios $\frac{M_{1}}{N}\;=\;\frac{t_{1}}{K_{1}}$, $\frac{M_{2}}{N}\;=\;\frac{t_{2}}{K_{2}}$ and transmission loads*

(4) R1=K1t1+1−K1−|LM|)t1+1K2t2+1/K1t1K2t2,R2=maxk1∈[K1]K2t2+1−K2−rank(D(k1))t2+1/K2t2.

Now, let us consider the performance of our two schemes. For the first layer, we claim that $R_{1}<\frac{1}{t_{2}+1}R_{\mathrm{base}1}$, where $t_{2}\geq 0$ since

R1Rbase1=K1t1+1−K1−|LM|)t1+1K2t2+1K1t1K2K1t1+1−K1−|LM|t1+1K1t1K2t2=K2t2+1K2K2t2=K2!t2!(K2−t2)!K2K2!(t2+1)!(K2−t2−1)!=t2!(K2−t2)(K2−t2−1)!K2t2!(t2+1)(K2−t2−1)!=K2−t2K2·1t2+1≤1t2+1.

Obviously, this scheme has the same performance as the Baseline Scheme, i.e., $R_{2}=R_{\mathrm{base}2}$, which also achieves the optimal transmission load when the demand matrix $D^{\left(k_{1}\right)}$, $k_{1}\;\in \;\left[K_{1}\right]$, is full rank.

**Figure 2:**
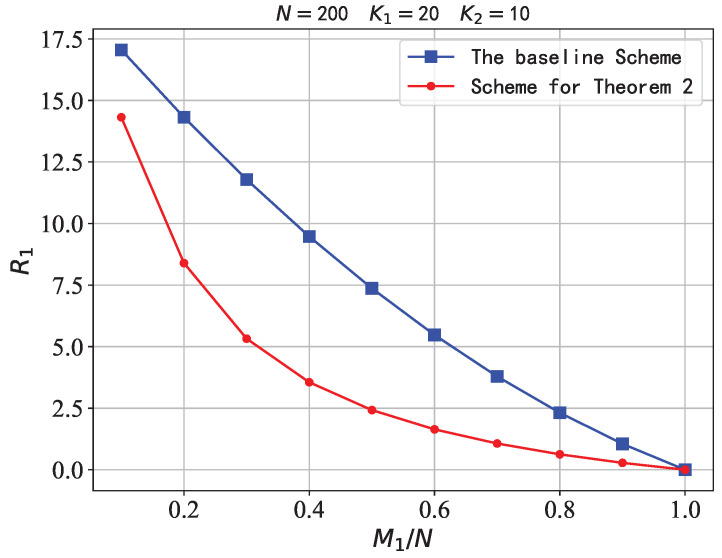
$R_{1}$ on N=200, $K_{1}\;=\;20$, $K_{2}\;=\;10$.

Finally, we perform a numerical comparison to further show the performance of our scheme. In Figure 2, we compare the Baseline Scheme with the scheme for Theorem 2 with fixed parameters $(K_{1},\;K_{2},\;|L_{M}|,\;N)\;=\;\left(20,\;10,\;10,\;200\right)$ and varying the memory ratio $M_{1}/N$ from 0 to 1 with a step size 0.1. As seen in Figure 2, compared to the Baseline Scheme, the scheme for Theorem 2 can reduce the transmission load $R_{1}$ significantly, as this scheme utilizes both the user’s cache and the mirror’s cache when constructing the multicast message sent by the server. The scheme for Theorem 2 achieves the same $R_{2}$ as the Baseline Scheme, while our scheme has a lower transmission load $R_{1}$.

## 4. Scheme for Theorem 2

In this section, we first give an illustrative example of our scheme. Then, the general description of the scheme is provided. Before the description, we first introduce the following lemmas regarding the message sent by the server and mirrors, whose proofs are included in Appendix A and Appendix B, respectively.

**Lemma** **1** (The messages sent by the server)**.** *Given a demand matrix D in *(1)*, the leader mirror set $L_{M}$, and a user set B∈([K2]t2+1), if there exists a mirror set C∈([K1]|LM|+t1+1), where $L_{M}\subseteq C$, let $V_{C}$ be the family of mirror set V, V⊆C, where each V satisfies Definition 1. Then, we have $\sum_{V\in V_{C}}X_{C\setminus V,B}\;=\;0$, where $X_{C\setminus V,B}$ represents the message sent by the server, which is defined in (8).*

**Lemma** **2** (The messages sent by the mirror)**.** *Given a sub-matrix $D^{\left(k_{1}\right)}$, $k_{1}\;\in \;\left[K_{1}\right]$ of D, the leader user set $L_{k_{1}}$, and a mirror set T1∈([K1]t1), if there exists a user set C′∈([K2]|Lk1|+t2+1), where $L_{k_{1}}\subseteq C^{\prime }$, let $V_{C^{\prime }}^{\prime }$ be the family of all set $V^{\prime }$, $V^{\prime }\subseteq C^{\prime }$, where each $V^{\prime }$ satisfies Definition 2. Then, we have $\sum_{V^{\prime }\in V_{C^{\prime }}^{\prime }}X_{T_{1},C^{\prime }\setminus V^{\prime }}^{\left(k_{1}\right)}\;=\;0$, where $X_{T_{1},C^{\prime }\setminus V^{\prime }}^{\left(k_{1}\right)}$ represents the message sent by the mirror $M_{k_{1}}$, which is defined in (9).*

By Lemma 1, for any mirror set A∈[K1]t1+1, $L_{M}⋂A\;=\;\emptyset$, and the message $X_{A,B}$, B∈[K2]t2+1 can be computed directly from the broadcast messages by using the following equation

(5) $$\begin{array}{c} X_{A,B}\;=\;\sum_{V\in V_{C}\setminus L_{M}}X_{C\setminus V,B} \end{array}$$

where $C\;=\;A⋃L_{M}$.

By Lemma 2, for any user set B=[K2]t2+1, $L_{k_{1}}⋂B\;=\;\emptyset$, the message $X_{T_{1},B}^{\left(k_{1}\right)}$ can be computed directly from the broadcast messages by using the equation

(6) $$\begin{array}{c} X_{T_{1},B}^{\left(k_{1}\right)}\;=\;\sum_{V^{\prime }\in V_{C^{\prime }}^{\prime }\setminus L_{k_{1}}}X_{T_{1},C^{\prime }\setminus V^{\prime }}^{\left(k_{1}\right)} \end{array}$$

where $C^{\prime }\;=\;B⋃L_{k_{1}}$. After receiving the messages sent by the mirror $M_{k_{1}}$, user U_*k*1,*k*2_ is able to recover its desired linear combination of files.

### 4.1. An Example for Theorem 2

When $K_{1}\;=\;3$, $K_{2}\;=\;2$, $t_{1}\;=\;t_{2}\;=\;1$, we can obtain an *F*-$\left(K_{1},K_{2};M_{1},M_{2};N\right)\;=\;6-\left(3,2;2,3;6\right)$ coded caching scheme as follows.

- **Placement phase**: Each file from $F_{2}^{B}$ is divided into 3121=6 subfiles with equal size, i.e., $W^{\left(n\right)}\;=\;\{W_{1,1}^{\left(n\right)}$, $W_{1,2}^{\left(n\right)},\ldots ,W_{3,1}^{\left(n\right)},W_{3,2}^{\left(n\right)}\}$, n∈[6]. For simplicity, we represent a set that is the subscript of some studied object by a string. For example, $T_{\left\{1,2\right\}}$ is represented by $T_{12}$. The contents cached by the mirrors are as follows:
  $$\begin{array}{c} Z_{1}\;=\;\left\{W_{1,1}^{\left(n\right)},W_{1,2}^{\left(n\right)}|\;n\;\in \;\left[6\right]\right\},Z_{2}\;=\;\left\{W_{2,1}^{\left(n\right)},W_{2,2}^{\left(n\right)}|\;n\;\in \;\left[6\right]\right\},Z_{3}\;=\;\left\{W_{3,1}^{\left(n\right)},W_{3,2}^{\left(n\right)}|n\;\in \;\left[6\right]\right\}. \end{array}$$
  The subfiles cached by the users are as follows:
  $$\begin{array}{c} {\tilde{Z}}_{1,1}\;=\;{\tilde{Z}}_{2,1}\;=\;{\tilde{Z}}_{3,1}\;=\;\left\{W_{1,1}^{\left(n\right)},W_{2,1}^{\left(n\right)},W_{3,1}^{\left(n\right)}|n\;\in \;\left[6\right]\right\},\\ {\tilde{Z}}_{1,2}\;=\;{\tilde{Z}}_{2,2}\;=\;{\tilde{Z}}_{3,2}\;=\;\left\{W_{1,2}^{\left(n\right)},W_{2,2}^{\left(n\right)},W_{3,2}^{\left(n\right)}|n\;\in \;\left[6\right]\right\}. \end{array}$$
- **Delivery phase**: In the delivery phase, the demand vectors with length 12 are $d_{k_{1},1}\;=\;\left(1,1,0,0,0,0\right),\;d_{k_{1},2}\;=\;\left(0,0,1,1,0,0\right),k_{1}\;\in \;\left[3\right]$. As we can see, $D^{\left(1\right)}\;=\;D^{\left(2\right)}\;=\;D^{\left(3\right)}$. Without loss of generality, we set $L_{M}\;=\;\left\{1\right\}$. We denote a linear combination of subfiles as
  $$L_{d_{k_{1},k_{2}}T_{1},T_{2}}\;=\;\sum_{n\in [N]}d_{k_{1},k_{2}}^{\left(n\right)}\cdot W_{T_{1},T_{2}}^{\left(n\right)}.$$
  where $T_{1}\;\in \;\left\{\left\{1\right\},\left\{2\right\},\left\{3\right\}\right\}$ and $T_{2}\;\in \;\left\{\left\{1\right\},\left\{2\right\}\right\}$, $k_{1}\;\in \;\left[3\right]$, $k_{2}\;\in \;\left[2\right]$. Then, the messages sent in this hierarchical system consist of the following two parts.
  – **The messages sent by the server:** The server generates signal $X_{A,B}$ satisfying A∈[3]2,B∈[2]2 and $A⋂L_{M}\;\neq \;⌀$ as follows:
    $$\begin{array}{cc} X_{12,12}\;=\; & L_{d_{1,1},2,2}⊕L_{d_{1,2},2,1}⊕L_{d_{2,1},1,2}⊕L_{d_{2,2},1,1}\\ \;=\; & \left({⊕}_{i\in [2]}\left(W_{2,2}^{\left(i\right)}⊕W_{1,2}^{\left(i\right)}\right)\right)⊕\left({⊕}_{i\in [3:4]}\left(W_{2,1}^{\left(i\right)}⊕W_{1,1}^{\left(i\right)}\right)\right),\\ X_{13,12}\;=\; & L_{d_{1,1},3,2}⊕L_{d_{1,2},3,1}⊕L_{d_{3,1},1,2}⊕L_{d_{3,2},1,1}\\ \;=\; & \left({⊕}_{i\in [2]}\left(W_{3,2}^{\left(i\right)}⊕W_{1,2}^{\left(i\right)}\right)\right)⊕\left({⊕}_{i\in [3:4]}\left(W_{3,1}^{\left(i\right)}⊕W_{1,1}^{\left(i\right)}\right)\right). \end{array}$$
    In this example, we have $L_{M}\;=\;\left\{1\right\}$, and A={2,3} has no intersection with $L_{M}$. Here, we have $C\;=\;L_{M}⋃A\;=\;\left\{1,2,3\right\}$ and $V_{C}\;=\;\left\{\left\{1\right\},\left\{2\right\},\left\{3\right\}\right\}$. By Lemma 2, we can generate $X_{23,12}\;=\;X_{12,12}+X_{13,12}\;=\;\left({⊕}_{i\in [2]}\left(W_{3,2}^{\left(i\right)}⊕W_{2,2}^{\left(i\right)}\right)\right)⊕\left({⊕}_{i\in [3:4]}\left(W_{3,1}^{\left(i\right)}⊕W_{2,1}^{\left(i\right)}\right)\right)$. Thus, the transmission load of the first layer is $R_{1}\;=\;\frac{3-1}{6}\;=\;1/3$.
  – **The messages sent by mirror $M_{k_{1}}$:** Here, we take mirror $M_{1}$ as an example. From $D^{\left(1\right)}$, we have $L_{1}\;=\;\left\{1,2\right\}$, and $M_{1}$ transmits $X_{T_{1},B}^{\left(1\right)}$, where T1∈[3]1, B∈[2]2, $B⋂L_{1}\;\neq \;\emptyset$, i.e.,
    $$\begin{array}{cc} X_{2,12}^{\left(1\right)}\; & =\;X_{12,12}-X_{1,12}^{\left(2\right)}\;=\;\left({⊕}_{i\in [2]}W_{2,2}^{\left(i\right)}\right)⊕\left({⊕}_{i\in [3:4]}W_{2,1}^{\left(i\right)}\right),\\ X_{3,12}^{\left(1\right)}\; & =\;X_{13,12}-X_{1,12}^{\left(3\right)}\;=\;\left({⊕}_{i\in [2]}W_{3,2}^{\left(i\right)}\right)⊕\left({⊕}_{i\in [3:4]}W_{3,1}^{\left(i\right)}\right),\\ X_{1,12}^{\left(1\right)}\; & =\;W_{1,2}^{\left(1\right)}⊕W_{1,2}^{\left(2\right)}⊕W_{1,1}^{\left(3\right)}⊕W_{1,1}^{\left(4\right)}. \end{array}$$
  Then, the transmission amount by mirror $M_{1}$ is 3 packets, and the transmission load of the second layer is $R_{2}\;=\;\frac{3}{6}\;=\;\frac{1}{2}$.

User $U_{1,1}$ can decode $W_{1,2}^{\left(1\right)}⊕W_{1,2}^{\left(2\right)}$, $W_{2,2}^{\left(1\right)}⊕W_{2,2}^{\left(2\right)}$, $W_{3,2}^{\left(1\right)}⊕W_{3,2}^{\left(2\right)}$, from $X_{1,12}^{\left(1\right)}$, $X_{2,12}^{\left(1\right)}$, $X_{3,12}^{\left(1\right)}$, respectively, as it has cached $\{W_{1,1}^{\left(n\right)},W_{2,1}^{\left(n\right)}$, $W_{3,1}^{\left(n\right)}\left|n\;\in \;\left[6\right]\right\}$.

Compared with the Baseline Scheme, which achieves $R_{\mathrm{base}1}\;=\;\frac{4}{3}$, $R_{\mathrm{base}2}\;=\;\frac{1}{2}$, our scheme has a significant improvement in $R_{1}$.

### 4.2. General Description of Scheme for Theorem 2

Given a $\left(K_{1},K_{2};M_{1},M_{2};N\right)$ hierarchical caching system, we have an *F*-$(K_{1},K_{2},M_{1}$, $M_{2},N)$ coded caching scheme where F=K1t1K2t2, $t_{1}\;\in \;\left[0:K_{1}\right]$, $t_{2}\;\in \;\left[0:K_{2}\right]$. The scheme consists of two phases.

- **Placement phase:** Firstly, we divide each file into K1t1 equal-size subfiles; then, we further divide each subfile into K2t2 sub-subfiles. The index of subfiles consists of two parts, $T_{1}$ and $T_{2}$, i.e., W(n)={WT1,T2(n)|T1∈[K1]t1,T2∈[K2]t2}, n∈[N]. Each mirror site $M_{k_{1}},k_{1}\;\in \;\left[K_{1}\right]$ caches subfiles $W_{T_{1},T_{2}}^{\left(n\right)}$ according to the following rule, which is mainly related to the first subscript $T_{1}$.
  Zk1=WT1,T2(n)|T1∈[K1]t1,T2∈[K2]t2,k1∈T1,n∈[N].
  Similarly, each user $U_{k_{1},k_{2}}$, $k_{1}\;\in \;\left[K_{1}\right],k_{2}\;\in \;\left[K_{2}\right]$ caches subfiles $W_{T_{1},T_{2}}^{\left(n\right)}$ according to the following rule, which is mainly related to the second subscript $T_{2}$.
  Z˜k1,k2=WT1,T2(n)|T1∈[K1]t1,T2∈[K2]t2,k2∈T2,n∈[N].
  Under this caching strategy, we can verify that it satisfies the memory constraints stated in Theorem 2. Each mirror caches K1−1t1−1K2t2N subfiles and each user caches K1t1K2−1t2−1N subfiles, where each subfile is B/K1t1K2t2 bits. Thus, the memory ratios of the mirror and user are $\frac{M_{1}}{N}\;=\;\frac{t_{1}}{K_{1}}$ and $\frac{M_{2}}{N}\;=\;\frac{t_{2}}{K_{2}}$, respectively. For any user’s demand vector $d_{k_{1},k_{2}}\;=\;\left(d_{k_{1},k_{2}}^{\left(1\right)},\ldots ,d_{k_{1},k_{2}}^{\left(N\right)}\right)$ of *N*-length, we use the notation as follows to denote a linear combination of subfiles:
  (7) Ldk1,k2T1,T2=∑n∈[N]dk1,k2(n)WT1,T2(n),T1∈[K1]t1,T2∈[K2]t2.
- **Delivery phase:** For the convenience of the subsequent discussion, we first give the following two definitions of the signals transmitted in the first layer, say $X_{A,B}$, and the second layer, say $X_{T_{1},B}^{\left(k_{1}\right)}$. For any mirror set containing $t_{1}+1$ mirrors defined as A∈[K1]t1+1, any mirror site set containing $t_{1}$ mirror sites defined as T1∈[K1]t1, and any user set containing $t_{2}+1$ users defined as B∈[K2]t2+1, we define
  (8) $$\begin{array}{cc} X_{A,B}\;=\; & \sum_{k_{1}\in A}\sum_{k_{2}\in B}L_{d_{k_{1},k_{2}},A\setminus \left\{k_{1}\right\},B\setminus \left\{k_{2}\right\}}, \end{array}$$
  (9) $$\begin{array}{cc} X_{T_{1},B}^{\left(k_{1}\right)}\;=\; & \sum_{k_{2}\in B}L_{d_{k_{1},k_{2}},T_{1},B\setminus \left\{k_{2}\right\}}. \end{array}$$
  After the demand matrix D of size $K_{1}K_{2}\times N$ and its sub-matrix $D^{\left(k_{1}\right)}$ of size $K_{2}\times N$ in (1) are revealed, we have the leader mirror set $L_{M}$ according to Definition 1. For each sub-matrix $D^{\left(k_{1}\right)}$ of D, $k_{1}\;\in \;\left[K_{1}\right]$, we have the leader user set $L_{k_{1}}$, $L_{k_{1}}\subseteq \left[K_{2}\right]$ according to Definition 2. There are two types of messages transmitted by the server and mirror, respectively.
  – **The messages sent by the server:** For each A∈[K1]t1+1, B∈[K2]t2+1, $L_{M}⋂A\;\neq \;\emptyset$, the server transmits $X_{A,B}$ to the mirror sites.
  – **The messages sent by the mirror:** Mirror site $M_{k_{1}}$ transmits $X_{T_{1},B}^{\left(k_{1}\right)}$ via the following rules.
    (1) For each T1∈[K1]t1, $k_{1}\;\notin \;T_{1}$, $A\;=\;T_{1}⋃\left\{k_{1}\right\}$, B∈[K2]t2+1, $B⋂L_{k_{1}}\;\neq \;\emptyset$, mirror $M_{k_{1}}$ transmits $X_{T_{1},B}^{\left(k_{1}\right)}$ by subtracting $\sum_{k_{1}^{\prime }\in T_{1}}X_{A\setminus \{k_{1}^{\prime }\},B}^{\left(k_{1}^{\prime }\right)}$ from $X_{A,B}$, i.e.,
    $$\begin{array}{c} X_{T_{1},B}^{\left(k_{1}\right)}=X_{A,B}-\sum_{k_{1}^{\prime }\in T_{1}}X_{A\setminus \{k_{1}^{\prime }\},B}^{\left(k_{1}^{\prime }\right)}. \end{array}$$
    (2) For each T1∈[K1]t1, $k_{1}\;\in \;T_{1}$, B∈[K2]t2+1, $B⋂L_{k_{1}}\;\neq \;\emptyset$, mirror $M_{k_{1}}$ directly transmits $X_{T_{1},B}^{\left(k_{1}\right)}$ to its connected users generated from its cached content $Z_{k_{1}}$.
  As regards the messages $X_{A,B}$, $A⋂L_{M}\;=\;\emptyset$, and $X_{T_{1},B}^{\left(k_{1}\right)}$, $B⋂L_{k_{1}}\;=\;\emptyset$, which are also necessary for the users, these messages can be computed from the sent messages by using Lemmas 1 and 2. More precisely, $X_{A,B}$, $A⋂L_{M}\;=\;\emptyset$ can be obtained by (5), and $X_{T_{1},B}^{\left(k_{1}\right)}$, $B⋂L_{k_{1}}\;=\;\emptyset$ can be obtained by (6).

Now, we prove that each message $X_{A,B}$ transmitted by the server is decodable, i.e., after each mirror subtracts some packets from $X_{A,B}$, the rest of the message only contains coded packets required by the users in $U_{k_{1}}$. Then, we further prove that each message $X_{T,B}^{\left(k_{1}\right)}$ transmitted by $M_{k_{1}}$ is decodable, i.e., after user U_*k*1,*k*2_, $k_{1}\;\in \;\left[K_{1}\right]$, $k_{2}\;\in \;\left[K_{2}\right]$, subtracting some packets from $X_{T,B}^{\left(k_{1}\right)}$, the rest of the message only contains coded packets required by user U_*k*1,*k*2_.

#### 4.2.1. Decodability of Mirror

For each mirror $M_{k_{1}}$, $k_{1}\;\in \;\left[K_{1}\right]$, it can receive or recover all the $X_{A,B}$$A\subseteq [K_{1}]$, $B\subseteq [K_{2}]$, from the server. By (8), we have

(10) $$\begin{array}{cc} X_{A,B}\;=\; & \sum_{k_{1}\in A}\sum_{k_{2}\in B}L_{d_{k_{1},k_{2}},A\setminus \left\{k_{1}\right\},B\setminus \left\{k_{2}\right\}}\\ \;=\; & \sum_{k_{1}\in A}\sum_{k_{2}\in B}\sum_{n\in [N]}d_{k_{1},k_{2}}^{\left(n\right)}W_{A\setminus \left\{k_{1}\right\},B\setminus \left\{k_{2}\right\}}^{\left(n\right)}\\ \;=\; & \sum_{k_{2}\in B}\sum_{n\in [N]}d_{k_{1},k_{2}}^{\left(n\right)}W_{A\setminus \left\{k_{1}\right\},B\setminus \left\{k_{2}\right\}}^{\left(n\right)} \end{array}$$

(11) $$\begin{array}{cc} \;\;\;\;\;\;\;\;\;\;\;\;\;\;\;+ & \sum_{k_{1}^{\prime }\in A\setminus \left\{k_{1}\right\}}\sum_{k_{2}\in B}\sum_{n\in [N]}d_{k_{1}^{\prime },k_{2}}^{\left(n\right)}W_{A\setminus \left\{k_{1}^{\prime }\right\},B\setminus \left\{k_{2}\right\}}^{\left(n\right)} \end{array}$$

$$\begin{array}{cc} \;\;\;\;\;\;\;\;\;\;\;\;\;\;\;\;\;\;\;\;\;\;\;\;\;\;\;\;\;\;= & \underset{\mathrm{The}\;\mathrm{coded}\;\mathrm{packets}\;\mathrm{required}\;\mathrm{by}\;\mathrm{users}\;\mathrm{in}\;U_{k_{1}}.}{\underbrace{X_{A\setminus \{k_{1}\},B}^{\left(k_{1}\right)}}}\\ \;\;\;\;\;\;\;\;\;\;\;\;\;\;+ & \underset{\mathrm{The}\;\mathrm{coded}\;\mathrm{packets}\;\mathrm{cached}\;\mathrm{by}\;M_{k_{1}}.}{\underbrace{\sum_{k_{1}^{\prime }\in A\setminus \left\{k_{1}\right\}}\sum_{k_{2}\in B}\sum_{n\in [N]}d_{k_{1}^{\prime },k_{2}}^{\left(n\right)}W_{A\setminus \left\{k_{1}^{\prime }\right\},B\setminus \left\{k_{2}\right\}}^{\left(n\right)}}} \end{array}$$

where (10) holds directly from (7), and (11) holds by separating $k_{1}$ from A. Moreover, (11) holds by (7) and (9). The first term of (11) denotes coded packets that will be transmitted to $U_{k_{1}}$ and the second term denotes packets cached by $M_{k_{1}}$ because $k_{1}\;\in \;A\setminus \left\{k_{1}^{\prime }\right\}$.

#### 4.2.2. Decodability of User

For each user $U_{k_{1},k_{2}}$, $k_{1}\;\in \;\left[K_{1}\right]$, $k_{2}\;\in \;\left[K_{2}\right]$, it can receive all the $X_{T_{1},B}^{\left(k_{1}\right)}$, $T_{1}\subseteq \left[K_{1}\right]$, $B\subseteq [K_{2}]$, $k_{2}\;\in \;B$, from mirror $M_{k_{1}}$. By (9), we have

(12) $$\begin{array}{ccc} X_{T_{1},B}^{\left(k_{1}\right)} & \;=\; & \sum_{k_{2}\in B}L_{d_{k_{1},k_{2}},T_{1},B\setminus \left\{k_{2}\right\}}\;=\;\underset{\;\mathrm{requested}\;\mathrm{by}\;\mathrm{user}\;U_{k_{1},k_{2}}}{\underbrace{\sum_{n\in [N]}d_{k_{1},k_{2}}^{\left(n\right)}W_{T_{1},B\setminus \left\{k_{2}\right\}}^{\left(n\right)}}} \end{array}$$

(13) $$\begin{array}{ccc} & + & \underset{\;\mathrm{cached}\;\mathrm{by}\;\mathrm{user}\;U_{k_{1},k_{2}}}{\underbrace{\sum_{k_{2}^{\prime }\in B\setminus \left\{k_{2}\right\}}\sum_{n\in [N]}d_{k_{1},k_{2}^{\prime }}^{\left(n\right)}W_{T_{1},B\setminus \left\{k_{2}^{\prime }\right\}}^{\left(n\right)}}}. \end{array}$$

where (12) holds directly by separating $k_{2}$ from B. It is clear that user U_*k*1,*k*2_ can decode its desired linear combination of packets, i.e., the first term of (12), by subtracting the cached contents, i.e., the second term of (12), as $k_{2}\;\in \;B\setminus \left\{k_{2}^{\prime }\right\}$, which means that U_*k*1,*k*2_ has already cached the packets from $X_{T_{1},B}^{\left(k_{1}\right)}$.

#### 4.2.3. Performance

From the placement phase, each file is firstly divided into K1t1 subfiles and then each subfile is further divided into K2t2 subfiles, so the subpacketization is K1t1K2t2. Each subfile is B/K1t1K2t2 bits, each mirror caches K1−1t1−1K2t2N subfiles, and each user caches K1t1K2−1t2−1N subfiles. Thus, the memory ratios of the mirror and user are $\frac{M_{1}}{N}\;=\;\frac{t_{1}}{K_{1}}$ and $\frac{M_{2}}{N}\;=\;\frac{t_{2}}{K_{2}}$, which satisfy the memory constraints in Theorem 2. In total, the server transmits K1t1+1−K1−|LM|t1+1K2t2+1 multicast messages, and the mirror transmits K2t2+1−K2−rank(D(k1))t1+1K1t1 multicast messages. Each message contains B/K1t1K2t2 bits, so the transmission loads of the first layer and the second layer are as illustrated in (4). Although the scheme for Theorem 2 has a higher subpacketization level of K1t1K2t2 compared with K1t1+K2t2 of the Baseline Scheme, we achieve a much lower transmission load $R_{1}$ under the same transmission load $R_{2}$, where both schemes achieve the optimal transmission load of the second layer when the sub-demand matrix $D^{\left(k_{1}\right)}$, $k_{1}\in \left[K_{1}\right]$ is full rank.

## 5. Conclusions

In this paper, we studied the linear function retrieval problem for hierarchical cache-aided networks. We proposed two schemes, where the first scheme achieves the optimal transmission load for the second layer for some demand distribution and our second scheme further reduces the load of the first layer while maintaining the same transmission load in the second layer.

## Data Availability

Data are contained within the article.

## Appendix A. Proof of Lemma 1

**Proof.** Without loss of generality, we assume that $L_{M}\;=\;[|L_{M}\left|\right]$. From (8) and (7), we have

(A1) $$\begin{array}{cc} \; & \sum_{V\in V_{C}}X_{C\setminus V,B}\;=\;\sum_{V\in V_{C}}\sum_{k_{1}\in C\setminus V}\sum_{k_{2}\in B}L_{d_{k_{1},k_{2}},C\setminus \left(V⋃\left\{k_{1}\right\}\right),B\setminus \left\{k_{2}\right\}}\\ \;=\; & \sum_{V\in V_{C}}\sum_{k_{1}\in C\setminus V}\sum_{k_{2}\in B}\sum_{n\in [N]}d_{k_{1},k_{2}}^{\left(n\right)}\cdot W_{C\setminus \left(V⋃\left\{k_{1}\right\}\right),B\setminus \left\{k_{2}\right\}}^{\left(n\right)}. \end{array}$$

If the occurrence number of each subfile $W_{T_{1},T_{2}}^{\left(n\right)}$, T1∈[K1]t1, T2∈[K2]t2, n∈[N] is even in $\sum_{V\in V_{C}}X_{C\setminus V,B}$, then the coefficient of $W_{T_{1},T_{2}}^{\left(n\right)}$ in the summation is 0. Note that if we focus on $W_{T_{1},T_{2}}^{\left(n\right)}$, then $k_{2}\;=\;B\setminus T_{2}$, which is a fixed user label. $W_{T_{1},T_{2}}^{\left(n\right)}$ appears in $\sum_{V\in V_{C}}X_{C\setminus V,B}$ if and only if there exist some mirrors $M_{k_{1}}$, $k_{1}\;\in \;C\setminus T_{1}$, which satisfy the two conditions: $d_{k_{1},k_{2}}^{\left(n\right)}\;\neq \;0,C\setminus \left(\left\{k_{1}\right\}⋃T_{1}\right)\;\in \;V_{C}$. Moreover, for each $k_{1}\;\in \;C\setminus T_{1}$ satisfying the two conditions, there exists one coded message that contains $W_{T_{1},T_{2}}^{\left(n\right)}$. Thus, we only need to prove that the number of mirrors $M_{k_{1}}$, $k_{1}\;\in \;C\setminus T_{1}$ satisfying the two conditions is even. Assume that mirror $k_{1}$ satisfies the two conditions; then, we have $d_{k_{1},k_{2}}^{\left(n\right)}\;\neq \;0$ and $C\setminus \left(\left\{k_{1}\right\}⋃T_{1}\right)\;\in \;V_{C}$. Let $L_{M}^{\prime }\;=\;C\setminus \left(\left\{x\right\}⋃T_{1}\right)\;=\;\left\{l_{1},\ldots ,l_{|L_{M}^{\prime }|}\right\}$, and $L_{M}^{\prime }$ is also a leader mirror set satisfying Definition 1. Then, by (2), we have $d_{k_{1},k_{2}}\;=\;\alpha_{1}^{\left(k_{1}\right)}d_{l_{1},k_{2}}+\ldots +\alpha_{|L_{M}^{\prime }|}^{\left(k_{1}\right)}d_{l_{|L_{M}^{\prime }|},k_{2}}$. Then, there must be $k_{1}^{\prime }$ mirrors in $\alpha_{1}^{\left(k_{1}\right)}d_{l_{1},k_{2}}+\ldots +\alpha_{|L_{M}^{\prime }|}^{\left(k_{1}\right)}d_{l_{|L_{M}^{\prime }|},k_{2}}$ satisfying $d_{k_{1},k_{2}}^{\left(n\right)}\;\neq \;0$ and the corresponding coefficient $\alpha_{l_{M}^{\prime }}^{\left(k_{1}^{\prime }\right)}\;\neq \;0$, $l_{M}^{\prime }\;\in \;[|L_{M}^{\prime }\left|\right]$. $k_{1}^{\prime }$ is an odd number; otherwise, $d_{k_{1},k_{2}}^{\left(n\right)}\;=\;0$. It is easy to check that these $k_{1}^{\prime }$ mirrors also satisfy constraints $d_{k_{1},k_{2}}^{\left(n\right)}\;\neq \;0,C\setminus \left(\left\{k_{1}\right\}⋃T_{1}\right)\;\in \;V_{C}$. Thus, there are in total $k_{1}^{\prime }+1$ mirrors in $C\setminus T_{1}$, which is an even number, satisfying the two constraints. □

## Appendix B. Proof of Lemma 2

**Proof.** Without loss of generality, we assume that $L_{k_{1}}\;=\;[|L_{k_{1}}\left|\right]$. From (9) and (7), we have

$$\begin{array}{cc} \; & \sum_{V^{\prime }\in V_{C^{\prime }}^{\prime }}X_{T_{1},C^{\prime }\setminus V^{\prime }}^{\left(k_{1}\right)}\;=\;\sum_{V^{\prime }\in V_{C^{\prime }}^{\prime }}\sum_{k_{2}\in C^{\prime }\setminus V^{\prime }}L_{d_{k_{1},k_{2}},T_{1},C^{\prime }\setminus \left(V^{\prime }⋃\left\{k_{2}\right\}\right)}\\ \;=\; & \sum_{V^{\prime }\in V_{C^{\prime }}^{\prime }}\sum_{k_{2}\in C^{\prime }\setminus V^{\prime }}\sum_{n\in [N]}d_{k_{1},k_{2}}^{\left(n\right)}\cdot W_{T_{1},C^{\prime }\setminus \left(V^{\prime }⋃\left\{k_{2}\right\}\right)}^{\left(n\right)}. \end{array}$$

If the occurrence number of subfile $W_{T_{1},T_{2}}^{\left(n\right)}$ is even in $\sum_{V^{\prime }\in V_{C^{\prime }}^{\prime }}X_{T_{1},C^{\prime }\setminus V^{\prime }}^{\left(k_{1}\right)}$, then the coefficient of $W_{T_{1},T_{2}}^{\left(n\right)}$ in the summation is 0. $W_{T_{1},T_{2}}^{\left(n\right)}$ appears in $\sum_{V^{\prime }\in V_{C^{\prime }}^{\prime }}X_{T_{1},C^{\prime }\setminus V^{\prime }}^{\left(k_{1}\right)}$ if and only if there exist some users U_*k*1,*x*_, $x\;\in \;C^{\prime }\setminus T_{2}$, which satisfy the following two conditions: $d_{k_{1},x}^{\left(n\right)}\;\neq \;0$ and $C^{\prime }\setminus \left(\left\{x\right\}⋃T_{2}\right)\;\in \;V_{C^{\prime }}^{\prime }$. Moreover, for each $x\;\in \;C^{\prime }\setminus T_{2}$ satisfying the two conditions, there exists one coded message that contains $W_{T_{1},T_{2}}^{\left(n\right)}$. Thus, we only need to prove that the number of users U_*k*1,*x*_, $x\;\in \;C^{\prime }\setminus T_{2}$ satisfying the two conditions is even. Assume that user U_*k*1,*x*_ satisfies the two conditions; then, we have $d_{k_{1},x}^{\left(n\right)}\;\neq \;0$ and $C^{\prime }\setminus \left(\left\{x\right\}⋃T_{2}\right)\;\in \;V_{C^{\prime }}^{\prime }$. Let $L_{k_{1}}^{\prime }\;=\;C^{\prime }\setminus \left(\left\{x\right\}⋃T_{2}\right)\;=\;\left\{l_{1},\ldots ,l_{|L_{k_{1}}^{\prime }|}\right\}$, and $L_{k_{1}}^{\prime }$ is also a leader user set among users in $U_{k_{1}}$, where $\mathrm{rank}\left(D^{\left(k_{1}\right)}\right)\;=\;\mathrm{rank}\left(D_{L_{k_{1}}^{\prime }}\right)$. Then, we have

$$d_{k_{1},k_{2}}\;=\;\alpha_{1}d_{k_{1},l_{1}}+\ldots +\alpha_{|L_{k_{1}}^{\prime }|}d_{k_{1},l_{|L_{k_{1}}^{\prime }|}},\left(\alpha_{1},\ldots ,\alpha_{|L_{k_{1}}^{\prime }|}\right)\;\in \;{\left[F_{2}\right]}^{|L_{k_{1}}^{\prime }|}.$$

Then, there must be $x^{\prime }$ users in $\alpha_{1}d_{k_{1},l_{1}}+\ldots +\alpha_{|L_{k_{1}}^{\prime }|}d_{k_{1},l_{|L_{k_{1}}^{\prime }|}}$, $\left(\alpha_{1},\ldots ,\alpha_{|L_{k_{1}}^{\prime }|}\right)\;\in \;{\left[F_{2}\right]}^{|L_{k_{1}}^{\prime }|}$ satisfying $d_{k_{1},x}^{\left(n\right)}\;\neq \;0$ and the corresponding coefficient $\alpha_{l_{k_{1}}^{\prime }}\;\neq \;0$, $k_{1}\;\in \;[|L_{k_{1}}^{\prime }\left|\right]$. $x^{\prime }$ is an odd number; otherwise, $d_{k_{1},x}^{\left(n\right)}\;=\;0$. It is easy to check that these $x^{\prime }$ users also satisfy $C^{\prime }\setminus \left(\left\{x\right\}⋃T_{2}\right)\;\in \;V_{C^{\prime }}^{\prime }$. Thus, there are in total $x^{\prime }+1$ users in $C^{\prime }\setminus T_{1}^{\prime }$, which is an even number, satisfying the two constraints. □
